# The Significance of the Prognostic Nutritional Index in Patients with Completely Resected Non-Small Cell Lung Cancer

**DOI:** 10.1371/journal.pone.0136897

**Published:** 2015-09-10

**Authors:** Shunsuke Mori, Noriyasu Usami, Koichi Fukumoto, Tetsuya Mizuno, Hiroaki Kuroda, Noriaki Sakakura, Kohei Yokoi, Yukinori Sakao

**Affiliations:** 1 Department of Thoracic Surgery, Aichi Cancer Center Hospital, 1–1 Kanokoden, Chikusa-ku, Nagoya, 464–8681, Japan; 2 Department of Thoracic Surgery, Nagoya University Graduate School of Medicine, 65 Tsurumai-cho, Showa-ku, Nagoya, 466–8550, Japan; Peking University People Hospital, CHINA

## Abstract

**Objectives:**

Immunological parameters and nutritional status influence the outcome of patients with malignant tumors. A prognostic nutritional index, calculated using serum albumin levels and peripheral lymphocyte count, has been used to assess prognosis for various cancers. This study aimed to investigate whether this prognostic nutritional index affects overall survival and the incidence of postoperative complications in patients with completely resected non-small cell lung cancer.

**Methods:**

We retrospectively reviewed the medical records of 409 patients with non-small cell lung cancer who underwent complete resection between 2005 and 2007 at the Aichi Cancer Center.

**Results:**

The 5-year survival rates of patients with high (≥50) and low (<50) prognostic nutritional indices were 84.4% and 70.7%, respectively (*p* = 0.0011). Univariate analysis showed that gender, histology, pathological stage, smoking history, serum carcinoembryonic antigen levels, and prognostic nutritional index were significant prognostic factors. Multivariate analysis identified pathological stage and the prognostic nutritional index as independent prognostic factors. The frequency of postoperative complications tended to be higher in patients with a low prognostic nutritional index.

**Conclusions:**

The prognostic nutritional index is an independent prognostic factor for survival of patients with completely resected non-small cell lung cancer.

## Introduction

Non-small cell lung cancer (NSCLC) has a poor prognosis and is one of the most common causes of cancer-related death worldwide [1]. It can be assessed using a number of prognostic factors including age, gender, tumor size, lymph node metastasis [2,3], smoking status [4,5], and serum carcinoembryonic antigen (CEA) level [6]. Furthermore, immunological parameters and nutritional status can influence disease outcome in patients with malignant tumors [7].

The European Lung Cancer Working Group [8] and the Japan Multinational Trial Organization [9] reported that an elevated neutrophil count was associated with poor prognosis in patients with NSCLC. The lymphocyte count has been reported to have independent prognostic significance in pancreatic cancer [10], breast cancer [11], and node-negative NSCLC [3]. Additionally, nutritional status, which is commonly evaluated using serum albumin levels, is an important prognostic factor in advanced cancer [12]. An elevated serum albumin level has been found to be associated with improved survival among patients with lung cancer [13].

The concept of a prognostic nutritional index (PNI) was suggested by Buzby and colleagues in 1980 [14]. PNI was proposed to assess prognostic factors in patients with malignant gastrointestinal tract tumors, liver cirrhosis [15], and chronic renal failure. Onodera and associates suggested that this PNI should be calculated using serum albumin levels and peripheral lymphocyte counts [16], and this was widely used as an indicator of nutritional status and to predict prognosis [12]. This PNI was found to be useful when predicting the prognosis of esophageal carcinoma [17], gastric carcinoma [7], pancreatic cancer [12], and hepatocellular carcinoma [18]. However, to the best of our knowledge, no studies till date have investigated the association between PNI and the prognosis of patients with completely resected NSCLC.

The present study aimed to investigate whether PNI can serve as an independent prognostic factor in patients with completely resected NSCLC.

## Materials and Methods

### Patients

The patient characteristics are presented in Table 1. This study comprised 542 patients surgically treated for primary lung cancer between 2005 and 2007 at the Aichi Cancer Center Hospital, Nagoya, Japan. Of these, 133 patients were excluded as they had unmeasured differential lymphocyte counts, incomplete resection, or insufficient data. This study was approved by the Institutional Review Board of Aichi Cancer Center.

**Table 1:** Patient Characteristics.

Characteristics		n (%)
Age (years)	median (range)	66 (32–86)
Gender	male	249 (60.9)
	female	160 (39.1)
Histology	AD	277 (67.7)
	SQ	88 (21.5)
	others	44 (10.8)
p-Stage	I	250 (61.1)
	II	81 (19.8)
	III	78 (19.1)
smoking history	yes	245 (60.3)
	no	161 (39.7)
CEA (ng/ml)	≤5	297 (73.5)
	>5	107 (26.5)
PNI	median (range)	51.4 (20.7–65.6)

AD, adenocarcinoma; CEA, carcinoembryonic antigen; n, number of patients; PNI, prognostic nutritional index; SQ, squamous cell carcinoma.

Because individual patients were not identified, our institutional review board approved this study without the requirement to obtain patient consent. The patient records were anonymized and de-identified prior to analysis. The following information was collected from the medical records and clinical database at our department: age at the time of surgery, gender, histology, pathological tumor-lymph node-metastasis (TNM) stage, smoking history, serum CEA level, postoperative complications, and survival. We calculated PNI using the following formula: PNI = serum albumin levels (g/dl) × 10 + total lymphocyte count (per mm^3^) × 0.005, as proposed by Onodera et al. [16]. Blood samples were collected from all patients 1 month prior to surgery, and pathological staging was recorded based on the seventh edition of the Union for International Cancer Control TNM classification [19]. Postoperative complications were defined according to the criteria of the Society of Thoracic Surgeons (STS) database [20]. The overall survival time was measured as the time elapsed between the date of surgery and the date of death or last follow-up.

### Statistical analysis

Survival curves were estimated using the Kaplan–Meier method, and differences in survival were assessed using the log-rank test. The receiver operating characteristics (ROC) curve of PNI was calculated to determine the optimal cut-off value. Univariate and multivariate analyses with a Cox proportional hazards model or logistic regression model were performed to assess significant factors. All statistical analyses were carried out using JMP version 10 statistical software (SAS Institute Inc., Cary, NC, USA), and *p* value < 0.05 was considered statistically significant.

## Results

**Fig 1:**
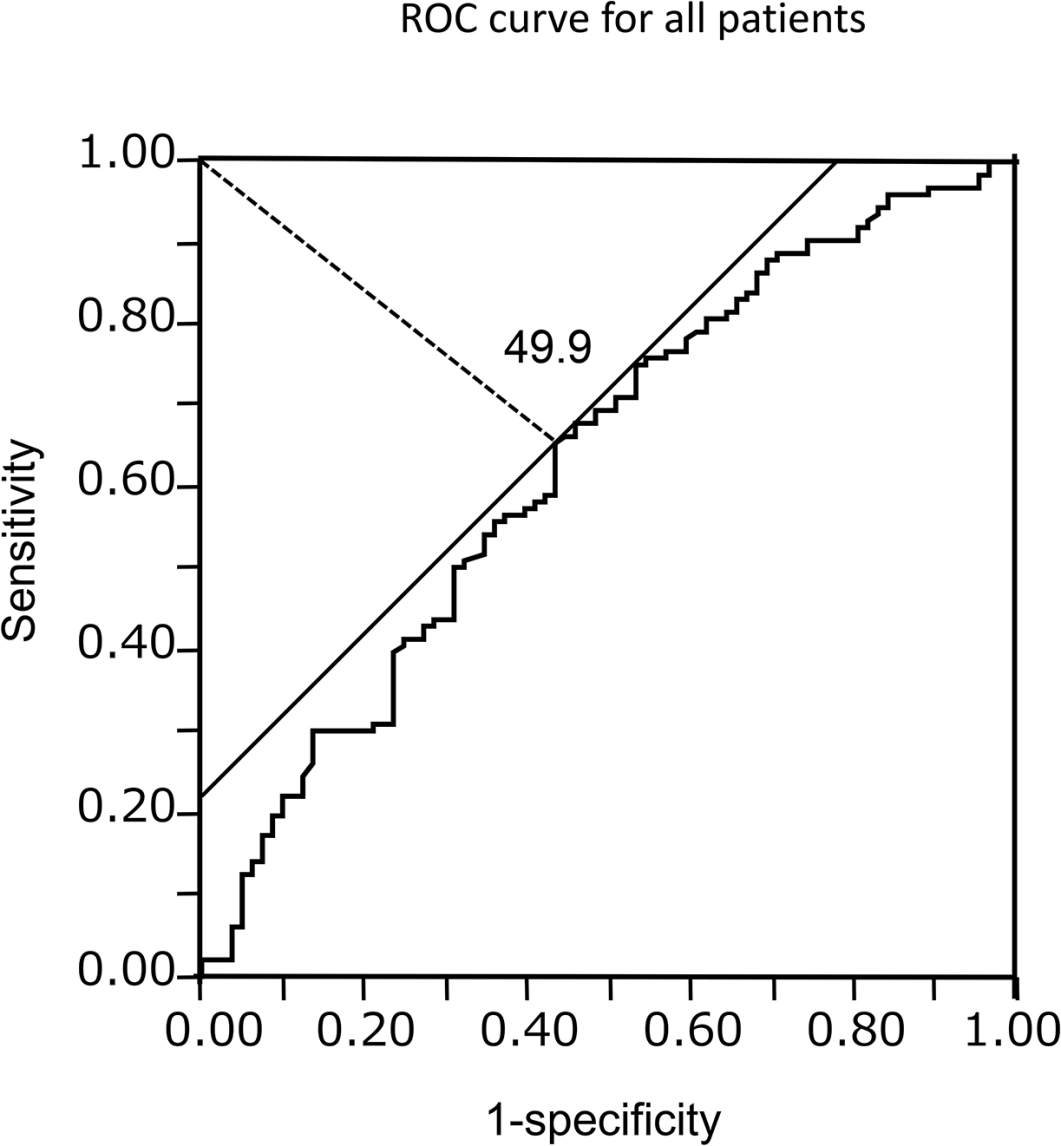
Receiver operating characteristic curve in all patients. Optimal cut-off for the prognostic nutritional index was 49.9 (Area under the curve = 0.63).

Between 2005 and 2007, 542 patients were surgically treated for primary lung cancer of which 133 patients were excluded from this study because of unmeasured differential lymphocyte count (n = 100), incomplete resection (n = 19), and insufficient data (n = 14). Thus, 409 patients [249 males and 160 females: median age: 66 years (range: 32–86 years)] were included in this study, and survivors were followed-up for a median of 55.1 months. The median PNI value was 51.4 (range: 20.7–65.6). The ROC curve identified an optimal cut-off value of 49.9 (Area under the curve = 0.63) (Fig 1), and based on this, the value for this study was fixed at 50.

**Fig 2:**
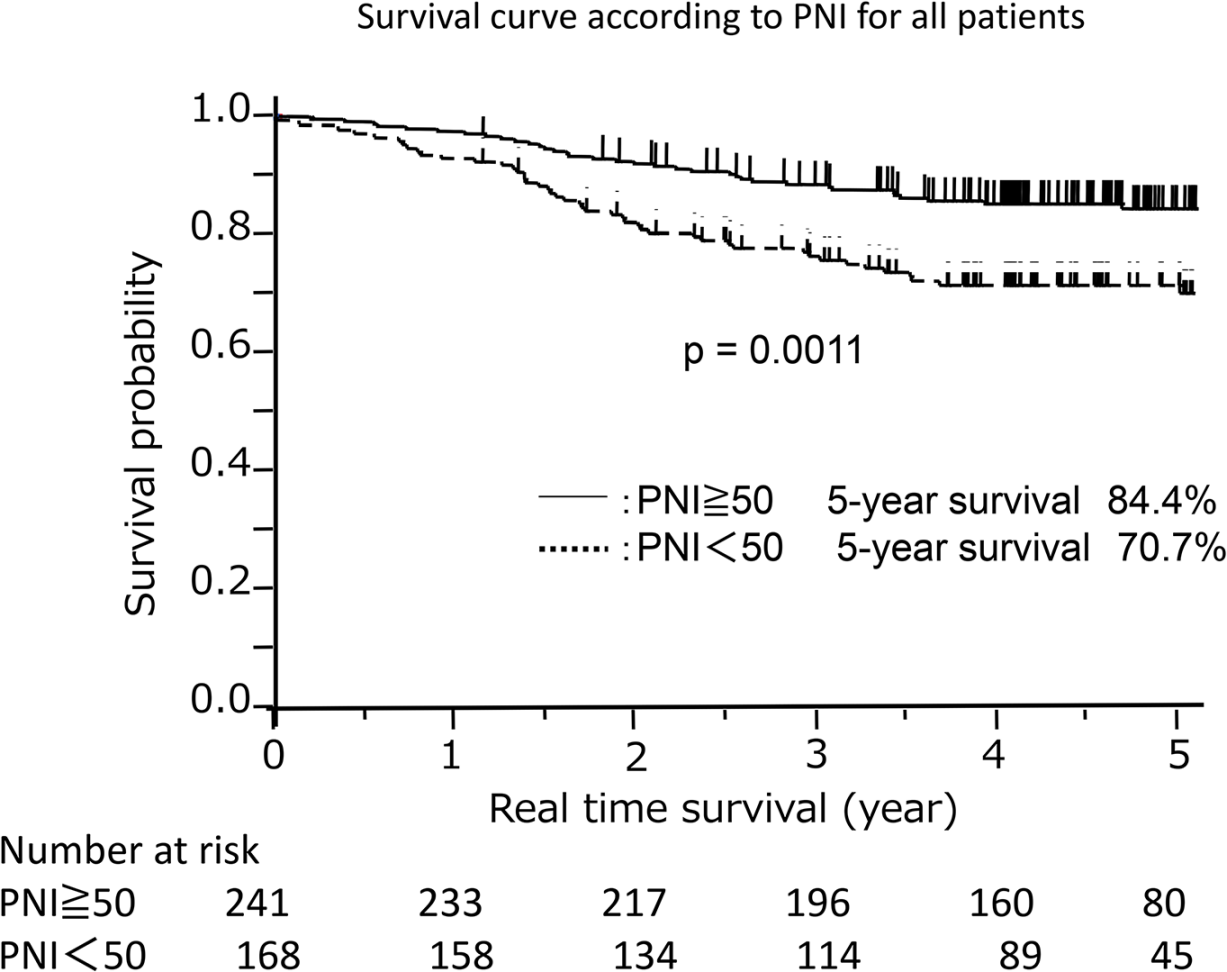
Survival curve according to the prognostic nutritional index. The survival of patients with a low prognostic nutritional index (< 50) was significantly poorer than that of patients with a high prognostic nutritional index (≥50).

Table 2 shows the relationship between PNI and the clinico-pathological features. Significant differences were observed with respect to age, gender, histology, and pathological stage. Patients with a higher PNI (≥50) tended to be younger females with adenocarcinoma and pathological stage I. Furthermore, the 5-year survival rates of patients with high (≥50) and low (<50) PNI were 84.4% and 70.7%, respectively (*p* = 0.0011, Fig 2).

**Table 2:** Relationship between PNI and clinico-pathological features.

	PNI ≥ 50 (n = 241)	PNI < 50 (n = 168)	p-value
Age			0.0013
<66	135 (56.0%)	67 (39.9%)	
≥66	106 (44.0%)	101 (60.1%)	
Sex			0.0047
male	133 (55.2%)	116 (69.0%)	
female	108 (44.8%)	52 (31.0%)	
Histology			0.0014
AD	180 (74.7%)	97 (57.7%)	
SQ	21 (8.7%)	23 (13.7%)	
others	40 (16.6%)	48 (28.6%)	
p-Stage			0.0102
I	162 (67.2%)	88 (52.4%)	
II	40 (16.6%)	41 (24.4%)	
III	39 (16.2%)	39 (23.2%)	

AD, adenocarcinoma; PNI, prognostic nutritional index; SQ, squamous cell carcinoma.

**Fig 3:**
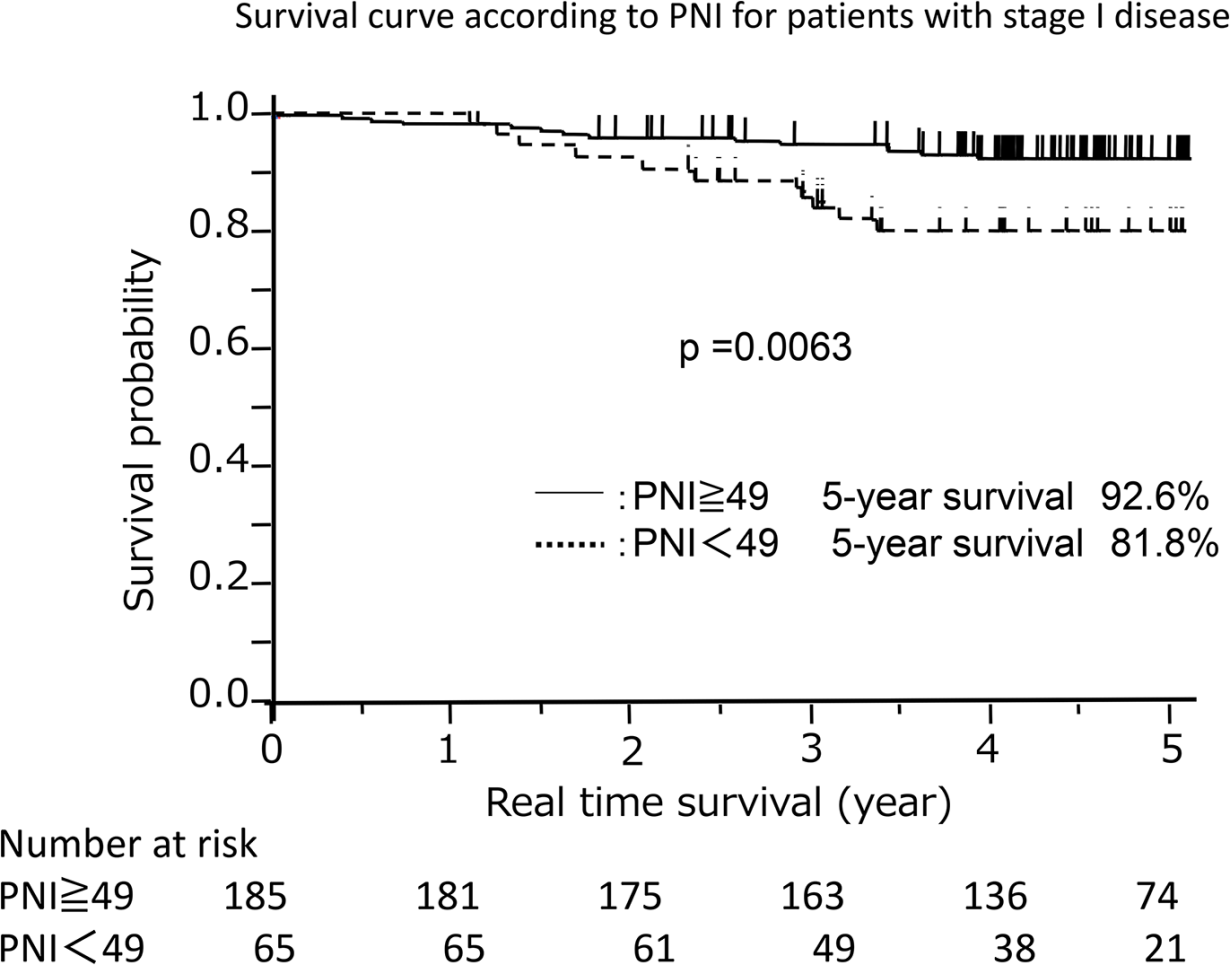
Survival curve according to the prognostic nutritional index in patients with pathological stage I disease. The survival of patients with a low prognostic nutritional index (< 49) was significantly different from those with a high prognostic nutritional index (≥49).

We also examined the relationship between PNI and prognosis in patients with pathological stage I disease. A PNI value of 49 corresponded to the optimal sensitivity and specificity on the ROC curve in patients with pathological stage I disease (Area under the curve = 0.62). Among these patients, the 5-year survival rates were significantly better in patients with high (≥49) PNI than in those with low (<49) PNI (92.6% versus 81.8%, respectively; *p* = 0.0063; Fig 3).

Univariate analysis showed that gender, histology, pathological stage, smoking history, serum CEA level, and PNI [low versus high; hazard ratio (HR) = 2.02; 95% confidence interval (CI) = 1.31–3.13; *p* = 0.0013] were significantly associated with poor survival. Multivariate analysis showed that PNI was an independent prognostic factor (low versus high; HR = 1.63; 95% CI = 1.04–2.57; *p* = 0.0310). Additionally, pathological stage was found to be an independent prognostic factor (Table 3).

**Table 3:** Univariate and multivariate analysis of overall survival.

			Univariate analysis		Multivariate analysis	
Characteristics		n	HR (95% CI)	p-value	HR (95% CI)	p-value
Age (years)	< 66	202	Reference			
	≥66	207	1.46 (0.95–2.26)	0.0876		
Gender	female	160	Reference		Reference	
	male	249	1.78 (1.12–2.93)	0.0148	1.22 (0.66–2.36)	0.5339
Histology	AD	277	Reference		Reference	
	non-AD	132	2.08 (1.35–3.19)	0.0011	1.03 (0.63–1.69)	0.8957
p-Stage	I	250	Reference		Reference	
	II	81	2.90 (1.61–5.18)	0.0005	2.32 (1.25–4.29)	0.0085
	III	78	7.06 (4.26–11.90)	< 0.0001	6.06 (3.52–10.61)	< 0.0001
Smoking history	no	161	Reference		Reference	
	yes	245	2.25 (1.39–3.80)	0.0008	1.30 (0.66–2.60)	0.4479
CEA (ng/ml)	≤5	297	Reference		Reference	
	> 5	107	2.02 (1.29–3.11)	0.0024	1.18 (0.74–1.87)	0.4738
PNI	≥50	241	Reference		Reference	
	< 50	168	2.02 (1.32–3.13)	0.0013	1.63 (1.04–2.56)	0.0322

AD, adenocarcinoma; CEA, carcinoembryonic antigen; CI, confidence interval; HR, hazard ratio; n, number of patients; PNI, prognostic nutritional index; SQ, squamous cell carcinoma.

We also investigated the correlation between PNI and postoperative complications. According to the collected STS data, 44 patients showed postoperative complications (Table 4); the most common ones being atrial arrhythmia requiring treatment and chylothorax requiring medical intervention. Univariate analysis showed that postoperative complications were associated with age ≥ 66 years, male gender, and non-adenocarcinoma. Moreover, patients with PNI < 50 showed an increased risk of postoperative complications, but this was not statistically significant (*p* = 0.0567, Table 5).

**Table 4:** Relationship between PNI and postoperative complications.

Complications	PNI ≥ 50 (n = 20)	PNI < 50 (n = 24)	total (n = 44)
Pulmonary			
Air leak > 5days duration	2	3	5
Atelectasis	0	1	1
Pleural effusion	1	2	3
Pneumonia	3	1	4
Pneumothorax	0	5	5
Other pulmonary event	1	3	4
Cardiovascular			
Atrial arrhythmia	5	2	7
Myocardial infarct	1	0	1
Infection			
Empyema	1	0	1
Surgical site infection	0	1	1
Neurology			
New central neurological event	1	0	1
Recurrent laryngeal nerve paresis	0	1	1
Delirium	2	1	3
Miscellaneous			
Chylothorax	3	4	7

PNI: prognostic nutritional index

Other pulmonary events include three cases of interstitial pneumonia and one case of chronic obstructive pulmonary disease exacerbation.

**Table 5:** Univariate analysis of postoperative complications.

Characteristics		N	HR	95% CI	p-value
Age (years)	< 66 (n = 202)	14	Reference		
	≥66 (n = 207)	30	2.28	1.19–4.56	0.0126
Gender	female (n = 160)	8	Reference		
	male (n = 249)	36	3.21	1.52–7.61	0.0016
Histology	AD (n = 277)	18	Reference		
	non-AD (n = 132)	26	3.52	1.86–6.78	0.0001
p-Stage	I (n = 250)	20	Reference		
	II (n = 81)	12	2	0.91–4.25	0.0838
	III (n = 78)	12	2.09	0.95–4.45	0.0669
Smoking history	no (n = 161)	15	Reference		
	yes (n = 245)	29	1.31	0.69–2.58	0.4205
CEA (ng/ml)	≤5 (n = 297)	30	Reference		
	> 5 (n = 107)	13	1.23	0.41–1.67	0.5606
PNI	≥50 (n = 241)	20	Reference		
	< 50 (n = 168)	24	1.84	0.98–3.49	0.0567

AD, adenocarcinoma; CEA, carcinoembryonic antigen; CI, confidence interval; HR, hazard ratio; N, number of patients showing postoperative complications; PNI, prognostic nutritional index.

## Discussion

The results of this study suggest that a low PNI is a poor independent prognostic factor for survival of patients with completely resected NSCLC. Although PNI can be used to predict the prognosis for various malignant tumors [7, 12, 17, 18], its relationship with completely resected NSCLC has not been previously defined. To the best of our knowledge, this is the first report regarding the usefulness of PNI in patients with completely resected NSCLC.

Buzby and colleagues [14] initially proposed PNI as a predictive indicator after digestive surgery. Their initial work used the following formula: PNI = 158 − 16.6 × albumin (g/100 ml) − 0.78 × triceps skinfold (mm) − 0.20 × transferrin (mg/100 ml) − 5.8 × cutaneous delayed hypersensitivity. In contrast, the PNI proposed by Onodera and coworkers [16] was calculated based on the serum albumin levels and total lymphocyte count, which are more readily assessable. Therefore, we used the latter technique in this study.

Several reports support using the lymphocyte count as a prognostic factor. Hespanhol and colleagues [21] reported that the lymphocyte count was a significant prognostic factor in patients with stage III or IV NSCLC, while Muers and associates [22] found that it was useful in patients with NSCLC who had not received curative treatment. Kobayashi and coworkers [3] demonstrated that the preoperative peripheral lymphocyte count was an independent prognostic factor in node-negative NSCLC. When the cut-off value for the lymphocyte count was 1900 cells/mm^3^, the overall survival rates were 67.9% and 87.7% for the low and high lymphocyte groups, respectively.

Lymphocytes play a fundamental role in cell-mediated immunity in various cancers, while inflammation plays an important role in NSCLC progression. Thus, the immune response to a tumor is lymphocyte dependent and as a result a low count can be a predictor of poor survival.

Serum albumin level is a simple surrogate marker for estimating protein levels, and it is commonly used as an indicator of nutritional status [12]. Gupta and colleagues [13] examined the association between pre-treatment serum albumin levels and survival of patients with different types of cancer. Of the 10 lung cancer studies reviewed, nine reported that higher serum albumin levels were associated with improved survival [13]. Therefore, pre-treatment serum albumin levels are useful prognostic indicators in cancer.

Onodera and coworkers [16] originally proposed PNI in 1984, and suggested that resection and anastomosis of the gastrointestinal tract could be performed safely in cases with a PNI > 45. The relationship between PNI and postoperative complications in patients with other malignant tumors has also been previously reported. Nozoe and associates [17] reported that the mean pre-operative PNI in esophageal cancer patients with postoperative complications was significantly lower than that in patients without postoperative complications. Kanda and colleagues [12] reported an association between low PNI and pancreatic fistulae in patients with pancreatic cancer. In the present study, we found no significant correlation between PNI and postoperative complications, although patients with a low PNI showed a tendency for increased risk of postoperative complications.

Our results also showed high 5-year survival rates, which can be explained by the fact that our cohort included a large number of patients with pathological stage I disease and adenocarcinoma. Our results were in accordance with the Japanese Lung Cancer Registry, which reported 5-year survival rates in patients with pathological stage I disease to be 74%–87% [23].

There were three major limitations to our study: (1) the retrospective study design, (2) the presence of inflammation was not evaluated, and (3) the degree of comorbidity was not accurately recorded. When a patient has an inflammatory condition, their serum albumin level and lymphocyte counts may be affected. Therefore, the absence of inflammation and comorbidity data may have potentially biased the results of our study. However, it is very important to know the factors affecting prognosis, and the role of PNI as a prognostic factor is noteworthy. We will prospectively use PNI to precisely evaluate therapeutic strategies.

In conclusion, the results of the present study demonstrate that PNI, calculated based on serum albumin level and lymphocyte count, is an independent prognostic factor for survival of patients with completely resected NSCLC and may serve as a useful prognostic tool.

## References

[1] Jemal A, Bray F, Center MM, Ferlay J, Ward E, Forman D. Global cancer statistics. CA Cancer J Clin. 2011;61:69–90. 10.3322/caac.20107 21296855

[2] Suzuki K, Nagai K, Yoshida J, Nishimura M, Takahashi K, Yokose T et al Conventional clinicopathologic prognostic factors in surgically resected nonsmall cell lung carcinoma. A comparison of prognostic factors for each pathologic TNM stage based on multivariate analyses. Cancer 1999;86:1976–1984. 10570421 10.1002/(sici)1097-0142(19991115)86:10<1976::aid-cncr14>3.0.co;2-i

[3] Kobayashi N, Usui S, Kikuchi S, Goto Y, Sakai M, Onizuka M et al Preoperative lymphocyte count is an independent prognostic factor in node-negative non-small cell lung cancer. Lung Cancer 2012;75:223–227. 10.1016/j.lungcan.2011.06.009 21764477

[4] Marugame T, Sobue T, Satoh H, Komatsu S, Nishino Y, Nakatsuka H et al Lung cancer death rates by smoking status: Comparison of the three-prefecture cohort study in Japan to the cancer prevention study II in the USA. Cancer Sci 2005;96:120–126. 15723657 10.1111/j.1349-7006.2005.00013.x PMC11158599

[5] Wakai K, Inoue M, Mizoue T, Tanaka K, Tsuji I, Nagata C et al Tobacco smoking and lung cancer risk: An evaluation based on a systematic review of epidemiological evidence among the Japanese population. Jpn J Clin Oncol 2006;36:309–324. 16735374 10.1093/jjco/hyl025

[6] Okada M, Nishio W, Sakamoto T, Uchino K, Yuki T, Nakagawa A et al. Prognostic significance of perioperative serum carcinoembryonic antigen in non-small cell lung cancer: Analysis of 1,000 consecutive resections for clinical stage I disease. Ann Thorac Surg 2004;78:216–221. 15223432 10.1016/j.athoracsur.2004.02.009

[7] Nozoe T, Ninomiya M, Maeda T, Matsukuma A, Nakashima H, Ezaki T. Prognostic nutritional index: A tool to predict the biological aggressiveness of gastric carcinoma. Surg Today 2010;40:440–443. 10.1007/s00595-009-4065-y 20425547

[8] Paesmans M, Sculier JP, Libert P, Bureau G, Dabouis G, Thiriaux J et al Prognostic factors for survival in advanced non-small-cell lung cancer: Univariate and multivariate analyses including recursive partitioning and amalgamation algorithms in 1,052 patients. The European Lung Cancer Working Party. J Clin Oncol 1995;13:1221–1230. 7738625 10.1200/JCO.1995.13.5.1221

[9] Teramukai S, Kitano T, Kishida Y, Kawahara M, Kubota K, Komuta K et al Pretreatment neutrophil count as an independent prognostic factor in advanced non-small-cell lung cancer: An analysis of Japan multinational trial organisation LC00-03. Eur J Cancer 2009;45:1950–1958. 10.1016/j.ejca.2009.01.023 19231158

[10] Fogar P, Sperti C, Basso D, Sanzari MC, Greco E, Davoli C et al Decreased total lymphocyte counts in pancreatic cancer: An index of adverse outcome. Pancreas 2006;32:22–28. 16340740 10.1097/01.mpa.0000188305.90290.50

[11] Ownby HE, Roi LD, Isenberg RR, Brennan MJ. Peripheral lymphocyte and eosinophil counts as indicators of prognosis in primary breast cancer. Cancer 1983;52:126–130. 6850535 10.1002/1097-0142(19830701)52:1<126::aid-cncr2820520123>3.0.co;2-y

[12] Kanda M, Fujii T, Kodera Y, Nagai S, Takeda S, Nakao A. Nutritional predictors of postoperative outcome in pancreatic cancer. Br J Surg 2011;98:268–274. 10.1002/bjs.7305 20960457

[13] Gupta D, Lis CG. Pretreatment serum albumin as a predictor of cancer survival: A systematic review of the epidemiological literature. Nutr J 2010;9:69 10.1186/1475-2891-9-69 21176210 PMC3019132

[14] Buzby GP, Mullen JL, Matthews DC, Hobbs CL, Rosato EF. Prognostic nutritional index in gastrointestinal surgery. Am J Surg 1980;139:160–167. 7350839 10.1016/0002-9610(80)90246-9

[15] Alvares-da-Silva MR, Reverbel da Silveira T. Comparison between handgrip strength, subjective global assessment, and prognostic nutritional index in assessing malnutrition and predicting clinical outcome in cirrhotic outpatients. Nutrition 2005;21:113–117. 15723736 10.1016/j.nut.2004.02.002

[16] Onodera T, Goseki N, Kosaki G. Prognostic nutritional index in gastrointestinal surgery of malnourished cancer patients. Nihon Geka Gakkai zasshi 1984;85:1001–1005 [Japanese]. 6438478

[17] Nozoe T, Kimura Y, Ishida M, Saeki H, Korenaga D, Sugimachi K. Correlation of pre-operative nutritional condition with post-operative complications in surgical treatment for oesophageal carcinoma. Eur J Surg Oncol 2002;28:396–400. 12099649 10.1053/ejso.2002.1257

[18] Pinato DJ, North BV, Sharma R. A novel, externally validated inflammation-based prognostic algorithm in hepatocellular carcinoma: The prognostic nutritional index (PNI). Br J Cancer 2012;106:1439–1445. 22433965 10.1038/bjc.2012.92 PMC3326674

[19] Goldstraw P, Crowley J, Chansky K, Giroux DJ, Groome PA, Rami-Porta R et al The IASLC lung cancer staging project: proposals for the revision of the TNM stage groupings in the forthcoming (seventh) edition of the TNM classification of malignant tumors. J Thorac Oncol 2007;2:706–14. 17762336 10.1097/JTO.0b013e31812f3c1a

[20] Cerfolio RJ, Bryant AS. Different diffusing capacity of the lung for carbon monoxide as predictors of respiratory morbidity. Ann Thorac Surg 2009;88:405–410. 10.1016/j.athoracsur.2009.04.015 19632384

[21] Hespanhol V, Queiroga H, Magalhaes A, Santos AR, Coelho M, Marques A. Survival predictors in advanced non-small cell lung cancer. Lung Cancer 1995;13:253–267. 8719065 10.1016/0169-5002(95)00497-1

[22] Muers MF, Shevlin P, Brown J. Prognosis in lung cancer: Physicians' opinions compared with outcome and a predictive model. Thorax 1996;51:894–902. 8984699 10.1136/thx.51.9.894 PMC472611

[23] Sawabata N, Miyaoka E, Asamura H, Nakanishi Y, Eguchi K, Mori M et al Japanese lung cancer registry study of 11,663 surgical cases in 2004: demographic and prognosis changes over decade. J Thorac Oncol. 2011;6:1229–1235. 10.1097/JTO.0b013e318219aae2 21610521

